# Antibiotic Use and Infection Patterns in Pediatric Tracheostomy Patients During Index Hospitalization

**DOI:** 10.1002/oto2.70290

**Published:** 2026-08-17

**Authors:** Romaine F. Johnson, Saudamini Lele, Alyssa C. Chapel, Cynthia S. Wang, Yann‐Fuu Kou, Stephen R. Chorney

**Affiliations:** ^1^ Department of Otolaryngology–Head & Neck Surgery University of Texas Southwestern Medical Center Dallas Texas USA; ^2^ Department of Pediatric Otolaryngology Children's Health Dallas Texas USA

**Keywords:** antimicrobial stewardship, antibiotic utilization, bacterial infections, pediatric tracheostomy

## Abstract

**Objective:**

To evaluate antibiotic utilization as a process measure in pediatric tracheostomy patients, establishing baseline variation across diagnostic categories to inform quality improvement and stewardship initiatives.

**Methods:**

We retrospectively studied pediatric tracheostomy patients at a tertiary children's hospital (2015‐2024). Antibiotic use was quantified across diagnostic categories, with prophylactic courses defined as 2 days or less. Cultures were reviewed to distinguish true bacterial infections from viral detections and colonization. Mixed‐effects logistic regression modeled infection risk, accounting for patient clustering. Sensitivity analyses excluded patients hospitalized over 365 days.

**Results:**

Of 429 patients (6.8 months [IQR 4.3‐55.9]), respiratory conditions were most common (43.8%), followed by cardiac (19.3%). Cardiac patients had the highest antibiotic exposure (mean 52.1 days) despite low infection rates (17.8%), while trauma patients had the highest infection rates (31.5%). Diagnostic category did not significantly affect infection risk after accounting for clustering. Only 19.6% of 38,030 cultures showed true bacterial infection (median time 14.1 days posttracheostomy). Viral detection cascades triggered 11.4% of bacterial cultures within 48 hours.

**Discussion:**

Antibiotic prescribing patterns often reflect institutional preferences rather than true infection risk. Individual patient factors more strongly predict infection than diagnostic category. This highlights the need for personalized stewardship and standardized viral detection protocols.

**Implications for Practice:**

High variability in antibiotic utilization across diagnostic categories represents a measurable stewardship target. Reducing unnecessary exposure may decrease length of stay, healthcare costs, and antibiotic‐associated complications.

Pediatric patients requiring tracheostomy represent one of the most medically complex inpatient populations, often experiencing prolonged hospitalizations, multiple comorbidities, and frequent febrile episodes.^1^, ^2^, ^3^, ^4^, ^5^, ^6^ These factors, coupled with heightened concern for serious infection, drive significant antibiotic utilization. In many cases, antibiotics are initiated empirically in response to fever or clinical change, even in the absence of confirmed infection, and may be continued for extended durations out of caution.^2^, ^7^, ^8^, ^9^, ^10^, ^11^

In quality reporting systems, any new antibiotic started after surgery is often flagged as a potential stewardship failure. For tracheostomy patients, this approach poses a problem.^7^, ^12^, ^13^, ^14^ Postoperative antibiotic courses may be brief, consistent with the intended surgical prophylaxis, or reflect empiric treatment in patients where the infection risk is perceived to be high.^15^, ^16^, ^17^, ^18^, ^19^, ^20^, ^21^, ^22^, ^23^

Despite these challenges, evidence to guide diagnostic‐specific stewardship approaches in pediatric tracheostomy patients remains limited. Patterns of antibiotic use before and after surgery, variation across diagnostic categories, and the relationship to microbiologic findings are poorly characterized. Recent studies have begun to assess these challenges; however, focusing on index hospitalization has remained understudied.^24^

The Children's Health Airway Management Program (CHAMP) provides a unique opportunity to address these gaps.^25^ As a multidisciplinary registry capturing all pediatric tracheostomy cases at a large tertiary center, CHAMP offers comprehensive data on patient characteristics, antibiotic utilization, and culture results. Building on prior work that described outcomes in this population,^7^, ^8^, ^25^, ^26^ we sought to map antibiotic prescribing patterns surrounding tracheostomy placement, quantify short‐ and long‐term postoperative courses, and examine the timing and microbiology of postsurgical cultures. The study aims to identify patterns relevant to both stewardship practice and the refinement of quality metrics in this high‐risk group. We hypothesize that antibiotic utilization varies substantially by diagnostic category independent of infection risk, representing a process measure amenable to stewardship intervention.

## Methods

### Study Population

This retrospective cohort study included all patients who underwent tracheostomy at Children's Medical Center Dallas between January 1, 2015, and December 31, 2024. Patients were automatically enrolled in the Children's Health Airway Management Program (CHAMP) registry on the day of tracheostomy placement. Full details of the program are described elsewhere. The study was approved by the University of Texas Southwestern Medical Center Institutional Review Board (STU‐2019‐1103). This study represents a baseline assessment of antibiotic utilization within the CHAMP registry to identify variation amenable to stewardship intervention.

Clinical diagnostic categories were defined using primary admission diagnosis codes: respiratory (including bronchopulmonary dysplasia, respiratory failure), cardiac (congenital heart disease, cardiomyopathy), other congenital (genetic syndromes, congenital anomalies), neuromuscular/central nervous system (cerebral palsy, spinal muscular atrophy), infectious (sepsis, pneumonia), oncologic (malignancy, oncologic treatment complications), trauma/injury, and other conditions.

### Antibiotic Utilization Measures

Total antibiotic exposure was calculated as the sum of all antibiotic days during the index hospitalization from tracheostomy to discharge. Prophylactic antibiotic use was defined as courses ≤2 days. Postsurgical antibiotic administration was captured as any antibiotic given after tracheostomy placement. Antibiotic utilization rates were calculated as the percentage of hospitalization days receiving antibiotics: (total antibiotic days/length of stay) × 100.

### Culture Analysis and Infection Classification

Postsurgical cultures were obtained after tracheostomy placement and classified by component type: viral respiratory pathogen panels, bacterial respiratory pathogen panels, and general microbiology cultures. To distinguish clinically meaningful bacterial infections from viral detections and colonization, we created a comprehensive infection classification system. Viral detections included positive results from respiratory pathogen panels targeting common viruses. True bacterial infections were defined as positive bacterial cultures, excluding normal respiratory flora.

Culture timing was calculated as the number of days from surgery to the first positive result, with negative values indicating preoperative cultures. The burden of resistant organisms was assessed by identifying methicillin‐resistant *Staphylococcus aureus* (MRSA) and *Pseudomonas aeruginosa* isolates from bacterial cultures. Viral detection cascades were identified when bacterial cultures were obtained within 48 hours of positive viral results, representing potential viral‐triggered diagnostic workups.

### Data Management

We categorized primary discharge ICD‐10 codes into eight mutually exclusive clinical groups using regular expression pattern matching. Overlapping antibiotic administrations were merged to calculate total exposure days, excluding duplicates and administrative errors. Temporal variables were validated for logical sequence and flagged for review. Extreme values for length of stay (>365 days) were used for sensitivity analysis. All time variables were converted to days, antibiotic class names standardized, and datasets linked via unique identifiers with match validation.

Cultures were obtained during clinical evaluation for suspected infection; clinical correlates such as fever, hypotension, and radiographic findings were not available for adjudication.

### Statistical Analysis

Continuous data were described using the mean with standard deviation or the median with interquartile range for continuous variables and counts with percentages for categorical variables. One‐way analysis of variance with the Bonferroni correction was used to test differences in prophylactic antibiotic duration across diagnostic categories. Multivariable linear regression was used to assess total antibiotic exposure, adjusting for age (classified by the American Academy of Pediatrics categories), diagnostic category, and length of stay, with robust standard errors.

Infection analysis utilized a hierarchical data structure, where multiple cultures (n = 38,030) were obtained from individual patients (n = 429). We used mixed‐effects logistic regression to model the risk of bacterial infection, accounting for patient‐level clustering by treating patients as random intercepts. Model calibration was assessed by comparing observed versus predicted rates across probability deciles, and discrimination was evaluated using the area under the receiver operating characteristic curve (AUC). Standard logistic regression with clustered standard errors was performed for comparison.

Chi‐square tests compared bacterial infection rates between diagnostic groups, with analysis restricted to true bacterial cultures to avoid bias from including viral detections. Culture cascade analysis examined the temporal relationship between viral detections and subsequent bacterial culture orders.

To assess the robustness of the findings, we performed a sensitivity analysis excluding patients with a length of stay greater than 365 days (the top 5th percentile) to evaluate whether the results were driven by extreme outliers. This sensitivity analysis was applied to both antibiotic utilization and infection prediction models. Missing data for regression analyses were handled using listwise deletion.

Statistical significance was set at *P* < .05. Statistics were performed using StataCorp 2025 *Stata Statistical Software: Release 19*. StataCorp LLC. This study adheres to *The Strengthening the Reporting of Observational Studies in Epidemiology (STROBE)* guidelines.

Additional details of the complete statistical analysis, including the updated bacterial infection modeling, are provided in the supplemental material titled *Complete Statistical Analysis‐Updated with Bacterial Infection Modeling*.

Datasets are not publicly available due to patient privacy considerations but are available from the corresponding author on reasonable request with appropriate institutional review board approval.

## Results

Between January 2015 and December 2024, 429 patients underwent tracheostomy and met the inclusion criteria. All records were complete for culture results and diagnostic categories therefore included in the final analysis. The median age at tracheostomy was median age 6.8 months (IQR: 4.3‐55.9), with 225 (52.4%) male patients. Respiratory conditions were the most common indication (188 patients, 43.8%), followed by cardiac diagnoses (83 patients, 19.3%). Most patients were infants at the time of surgery (260 patients, 60.6%). The cohort was racially diverse, with 236 (55.0%) White, 144 (33.6%) Black or African American, and 144 (33.6%) Hispanic patients. Complete demographic characteristics are presented in Table 1.

**Table 1:** Demographics and Clinical Characteristics of 429 Pediatric Tracheostomy Patients, 2015 to 2024

	Summary
**N**	429
Patient age, months	6.8 (43)
Length of Stay	135.3 (103.5)
ICU days	118.4 (97.4)
Post Trach LOS	75.2 (74.9)
RACE	
American Indian and Alaska Native	6 (1.4%)
Asian	16 (3.7%)
Black or African American	144 (33.6%)
Hispanic	2 (0.5%)
Native Hawaiian and Other Pacific Islander	3 (0.7%)
Other	21 (4.9%)
Unknown	1 (0.2%)
White or Caucasian	236 (55.0%)
Ethnicity	
Hispanic	144 (33.6%)
Non‐Hispanic	283 (66.0%)
Unknown	2 (0.5%)
Sex	
Female	204 (47.6%)
Male	225 (52.4%)
AAP Age Categories	
Infant (<1 y)	260 (60.6%)
Toddler (1‐2 y)	41 (9.6%)
Preschool (3‐4 y)	23 (5.4%)
School‐age (5‐11 y)	51 (11.9%)
Adolescent (12‐17 y)	48 (11.2%)
Young adult (≥18 y)	6 (1.4%)
Clinical diagnostic categories	
Respiratory	188 (43.8%)
Cardiac	83 (19.3%)
Other congenital	37 (8.6%)
Neuromuscular/CNS	34 (7.9%)
Infectious	25 (5.8%)
Oncologic	13 (3.0%)
Trauma/injury	17 (4.0%)
Other	32 (7.5%)

Continuous variables: mean (SD); categorical variables.

Abbreviations: AAP, American Academy of Pediatrics; CNS, central nervous system; ICU, intensive care unit; LOS, length of stay.

**Figure 1:**
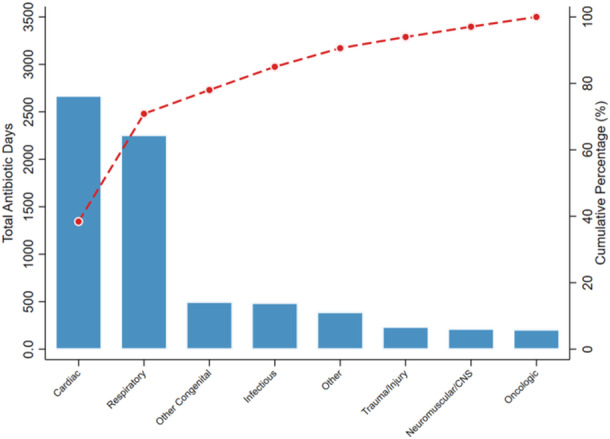
Pareto analysis of antibiotic utilization by diagnostic category. Bars represent total antibiotic days; line shows cumulative percentage. Cardiac and respiratory categories account for approximately 70% of total antibiotic exposure.

Table 2 shows antibiotic utilization among the 3303 antibiotic courses. Cardiac patients received the highest total antibiotic exposure (mean 52.1 days, SD 35.6) and had the longest patient‐days (mean 292.6 days, SD 177.7), while neuromuscular/CNS patients had the lowest exposure (mean 11.5 days, SD 9.6). Despite longer total exposure, infectious disease patients had the highest antibiotic utilization rate at 31.7% of hospitalization days compared to 10.9% for neuromuscular/CNS patients (*P* < .001). Prophylactic antibiotic courses (≤2 days) were administered in 73.8% of cases, with significant variation by diagnostic category (*P *< 0.001). These findings reveal substantial differences in antibiotic prescribing practices across patient populations. See Figure 1 for Pareto chart.

**Table 2:** Antibiotic Use by Diagnostic Category Among 3303 Antibiotic Courses

Variable	Resp	Card	Other cong	Neuro	Infect	Onc	Trauma	Other	Total	*P*‐value
N (%)	954 (28.9)	1,378 (41.7)	233 (7.1)	95 (2.9)	258 (7.8)	86 (2.6)	72 (2.2)	227 (6.9)	3,303 (100)	
DOT										<.001
Mean (SD)	24.8 (20.5)	52.1 (35.6)	46.7 (45.8)	11.5 (9.6)	35.4 (26.9)	21.6 (10.9)	17.4 (13.4)	23.3 (15.8)	37.8 (32.7)	
Patient‐days										<.001
Mean (SD)	167.2 (91.5)	292.6 (177.7)	279.2 (179.0)	122.5 (78.0)	134.5 (85.9)	185.0 (122.1)	93.9 (67.8)	210.5 (114.6)	225.8 (156.3)	
Abx classes										<.001
Mean (SD)	3.4 (1.1)	4.2 (1.0)	3.9 (1.2)	2.3 (0.7)	3.7 (0.9)	3.3 (0.8)	2.8 (0.8)	4.4 (1.2)	3.8 (1.1)	
% LOS on Abx										<.001
Mean (SD)	14.9 (13.0)	18.6 (7.3)	15.3 (12.3)	10.9 (7.9)	31.7 (29.8)	15.6 (14.6)	21.1 (15.7)	12.0 (8.3)	17.5 (13.5)	
Antibiotic class used, n (%)										
Aminoglycosides	94 (10.2)	42 (3.1)	25 (11.0)	3 (3.4)	6 (2.3)	3 (3.5)	3 (4.3)	17 (7.7)	193 (5.9)	<.001
Anti‐MRSA	221 (24.0)	586 (42.6)	64 (28.2)	29 (33.0)	126 (48.8)	34 (40.0)	25 (35.7)	79 (35.7)	1,164 (35.9)	
Carbapenems	21 (2.3)	49 (3.6)	4 (1.8)	0 (0.0)	19 (7.4)	2 (2.4)	1 (1.4)	6 (2.7)	102 (3.1)	
Cephalosporins	341 (37.1)	394 (28.6)	57 (25.1)	39 (44.3)	69 (26.7)	28 (32.9)	22 (31.4)	62 (28.1)	1,012 (31.2)	
Macrolides	8 (0.9)	6 (0.4)	0 (0.0)	0 (0.0)	1 (0.4)	0 (0.0)	2 (2.9)	2 (0.9)	19 (0.6)	
Other	38 (4.1)	119 (8.6)	7 (3.1)	2 (2.3)	10 (3.9)	5 (5.9)	3 (4.3)	18 (8.1)	202 (6.2)	
Penicillins	153 (16.6)	115 (8.4)	53 (23.3)	9 (10.2)	20 (7.8)	12 (14.1)	11 (15.7)	23 (10.4)	396 (12.2)	
Lincosamides	24 (2.6)	26 (1.9)	3 (1.3)	2 (2.3)	2 (0.8)	0 (0.0)	2 (2.9)	7 (3.2)	66 (2.0)	
Monobactams	1 (0.1)	0 (0.0)	2 (0.9)	0 (0.0)	0 (0.0)	0 (0.0)	0 (0.0)	0 (0.0)	3 (0.1)	
Nitroimidazoles	19 (2.1)	39 (2.8)	12 (5.3)	4 (4.5)	5 (1.9)	1 (1.2)	1 (1.4)	7 (3.2)	88 (2.7)	
Prophylaxis ≤ 2 d										<.001
Yes, n (%)	628 (65.8)	1,092 (79.2)	170 (73.0)	66 (69.5)	206 (79.8)	54 (62.8)	46 (63.9)	176 (77.5)	2438 (73.8)	
Post‐op Abx										.058
Yes, n (%)	387 (40.6)	533 (38.7)	78 (33.5)	31 (32.6)	89 (34.5)	36 (41.9)	21 (29.2)	73 (32.2)	1248 (37.8)	

Continuous variables: mean (SD); categorical variables: n (%). *P*‐values from regression (continuous) or Pearson chi‐square (categorical).

Abbreviations: Abx, antibiotic; Card, cardiac; DOT, days of therapy; Infect, infectious; LOS, length of stay; MRSA, methicillin‐resistant *Staphylococcus aureus*; Neuro, neuromuscular/central nervous system; Onc, oncologic; Other Cong, other congenital; Resp, respiratory.

After adjusting for age, diagnostic category, and length of stay, significant associations with total antibiotic exposure remained (Table 3). Compared to respiratory patients, cardiac patients received 10.6 additional antibiotic days (95% CI: 9.1‐12.2, *P* < .001), while neuromuscular/CNS patients received 6.1 fewer days (95% CI: −8.7 to −3.4, *P* < .001). Age demonstrated independent effects, with young adults (≥18 years) receiving 21.1 additional days compared to infants (95% CI: 14.2‐28.1, *P* < .001). The length of stay was strongly associated with antibiotic exposure (coefficient 0.17 days per hospital day, *P* < .001). The multivariable model confirms that diagnostic category independently influences antibiotic utilization beyond patient age and hospitalization complexity.

**Table 3:** Multivariable Linear Regression—Total Antibiotic Exposure

Variable	Coefficient	SE	95% CI	*P*‐value
Age category (ref: Infant)				
Toddler (1‐2 y)	9.31	1.11	7.14, 11.48	<.001
Preschool (3‐4 y)	15.82	3.47	9.01, 22.63	<.001
School‐age (5‐11 y)	5.54	1.15	3.28, 7.80	<.001
Adolescent (12‐17 y)	10.67	1.23	8.27, 13.07	<.001
Young adult (=18 y)	21.15	3.56	14.17, 28.13	<.001
Diagnostic category (ref: Respiratory)				
Cardiac	10.64	0.79	9.10, 12.19	<.001
Other congenital	7.94	1.52	4.96, 10.92	<.001
Neuromuscular/CNS	−6.06	1.33	−8.67, −3.45	<.001
Infectious	8.04	1.88	4.36, 11.72	<.001
Oncologic	−10.05	1.73	−13.45, −6.66	<.001
Trauma/injury	−0.51	1.82	−4.08, 3.05	.778
Other	−6.30	1.24	−8.73, −3.88	<.001
Hospital factors				
Length of stay (days)	0.17	0.003	0.16, 0.17	<.001
Constant	−7.94	0.66	−9.24, −6.64	<.001

Model statistics: N = 3096; *R* ^2^ = 0.70; *F* (13,308) = 579.31; *P* < .001; Root MSE = 18.23.

Multivariable regression of total antibiotic days. Robust standard errors used.

Abbreviation: CNS, central nervous system.

Postsurgical culture analysis included 38,030 cultures from 429 patients, encompassing viral respiratory pathogen panels, bacterial respiratory panels, and general microbiology cultures. When all culture types were included, 82.7% demonstrated positive results. However, this high rate reflected the inclusion of viral detections (44.8% of all cultures) and predetermined positive bacterial respiratory panels (16.0%), which are inherently positive. True bacterial infections, identified through general microbiology cultures, occurred in 19.6% of cultures overall (Table 4).

**Table 4:** Culture Analysis and Bacterial Infection Outcomes by Diagnostic Category

Diagnostic category	N cultures	True bacterial infection, n (%)	Median days to infection (IQR)
Trauma/Injury	664	209 (31.5%)	2.1 (40.8)
Oncologic	1178	279 (23.7%)	10.3 (82.0)
Respiratory	13,916	3,180 (22.9%)	14.9 (76.6)
Neuromuscular/CNS	2006	415 (20.7%)	18.8 (62.0)
Cardiac	11,940	2,128 (17.8%)	18.0 (106.2)
Other	1502	261 (17.4%)	9.2 (105.1)
Infectious	3497	576 (16.5%)	4.7 (61.0)
Other Congenital	3327	414 (12.4%)	14.5 (57.5)
Total	38,030	7,462 (19.6%)	14.1 (80.2)

Sorted by infection rate descending.

Abbreviations: CNS, central nervous system; IQR, interquartile range.

Bacterial infection rates varied by diagnostic category when viral detections were excluded (*χ* ^2^ = 324.9, *P* < .001). Trauma/injury patients demonstrated the highest bacterial infection rates (31.5%), followed by oncologic (23.7%) and respiratory patients (22.9%). Cardiac patients showed the lowest bacterial infection rates (17.8%) among all groups, despite receiving the highest antibiotic exposure. Other congenital patients had the lowest infection rates (12.4%). The median time to first bacterial infection was 14.1 days posttracheostomy (IQR: 80.2; range: −30 to 365 days). Resistant organisms were uncommon among bacterial cultures, with MRSA identified in 239 (0.6%) cultures (0.6%) and *P. aeruginosa* in 75 (0.2%) isolates.

Viral detection cascade analysis revealed that 11.4% of bacterial cultures were obtained within 48 hours of positive viral results. This cascade effect varied by diagnostic category, with other congenital patients showing the highest rate (17.9%) and trauma patients the lowest (6.7%).

Since multiple cultures were performed per patient (mean, 96.7 cultures per patient; range, 2‐914), we used a mixed‐effects logistic regression model to account for patient‐level clustering and assess the risk of bacterial infection (Table 5). The mixed‐effects model demonstrated significant patient‐level heterogeneity (random effect variance 3.20, *P* < .001). When properly accounting for clustering, most diagnostic category effects observed in standard analysis lost statistical significance. Time to culture remained the strongest predictor (OR 1.002, 95% CI: 1.001‐1.002, *P* < .001), while adolescent age showed a marginal association with a higher infection risk (OR 1.92, 95% CI: 0.96‐3.84, *P* = .065).

**Table 5:** Mixed‐Effects Logistic Regression: Predictors of Bacterial Infection

Variable	Odds ratio	95% Confidence interval	*P*‐value
Diagnostic category (ref: Respiratory)			
Cardiac	0.63	0.38‐1.06	.079
Other congenital	0.51	0.24‐1.09	.083
Neuromuscular/CNS	0.86	0.39‐1.91	.713
Infectious	0.91	0.40‐2.04	.811
Oncologic	0.63	0.19‐2.04	.436
Trauma/injury	0.60	0.20‐1.77	.352
Other	0.47	0.20‐1.10	.080
Age category (ref: Infant <1 y)			
Toddler (1‐2 y)	1.41	0.71‐2.80	.322
Preschool (3‐4 y)	0.95	0.39‐2.28	.906
School‐age (5‐11 y)	0.74	0.38‐1.43	.368
Adolescent (12‐17 y)	1.92	0.96‐3.84	.065
Young adult (≥18 y)	0.60	0.12‐3.13	.545
Time to culture (per day)	1.002	1.001‐1.002	<.001

Random effect variance = 3.20, 2.58‐3.9, <0.001; Log‐likelihood = −16,437.9; LR test vs standard logistic *χ* ^2 ^= 4245.4, *P* < .001. Discrimination (AUC) = 0.755, 95% CI (0.749‐0.761). Mixed‐effects logistic regression with patients as random intercepts. Robust standard errors used.

Abbreviations: AUC, area under receiver operating characteristic curve; CNS, central nervous system.

**Figure 2:**
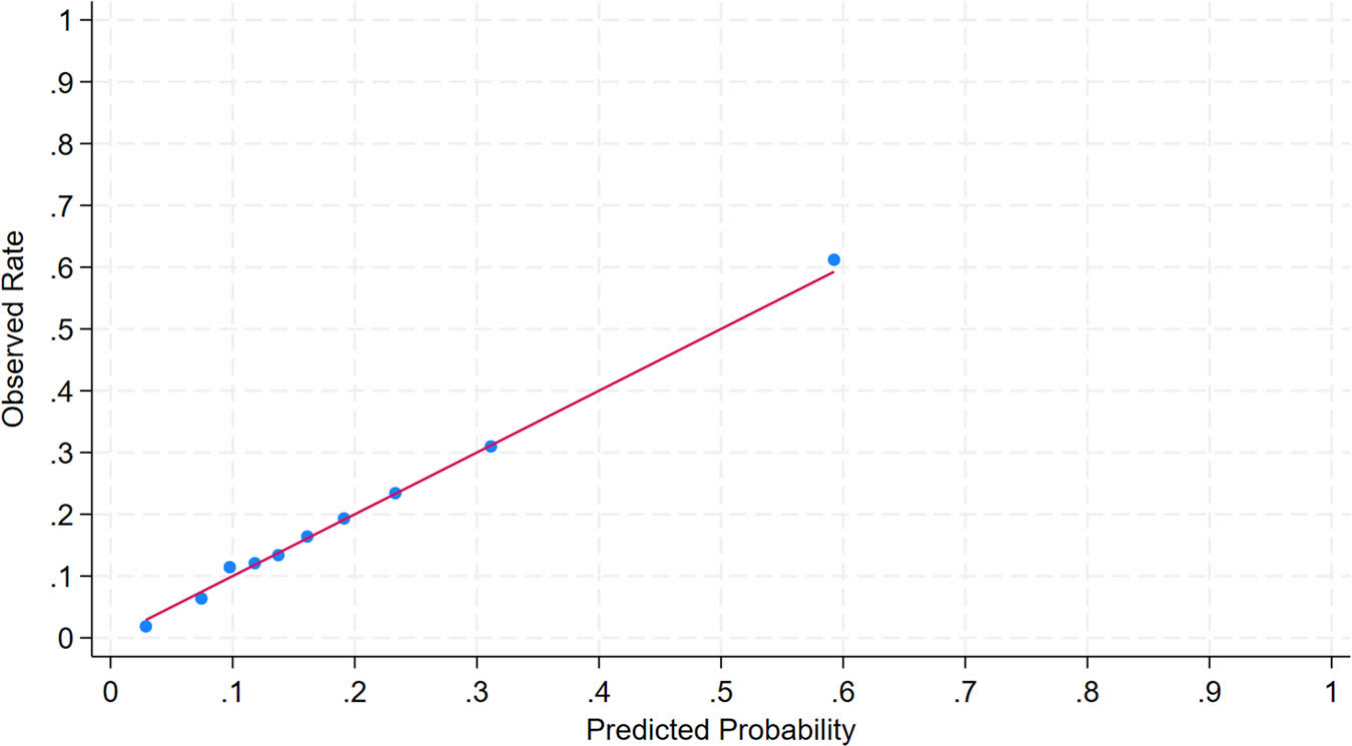
Model calibration plot. Observed bacterial infection rates (*y*‐axis) are plotted against model‐predicted probabilities (*x*‐axis) across probability deciles. The dashed diagonal line represents perfect calibration, where predicted and observed rates are equal. Points falling near this line indicate accurate risk estimation. The mixed‐effects logistic regression model demonstrated good discrimination with an area under the receiver operating characteristic curve (AUC) of 0.755 (95% CI: 0.749‐0.761), indicating reliable separation between cultures with and without true bacterial infection.

Regression model calibration analysis showed that the observed infection rates closely matched the predicted probabilities, indicating that the model provides clinically meaningful risk estimates (see Figure 2). The area under the receiver operating characteristic curve of 0.755 (95% CI: 0.749‐0.761) demonstrated good ability to distinguish between cultures with and without bacterial infection.

Excluding 671 antibiotic courses from patients with a length of stay >365 days, the sensitivity analysis included 2632 courses and confirmed the primary findings. The cardiac versus respiratory difference in antibiotic exposure remained significant but decreased from 27.4 to 17.9 additional days (*P* < .001). Prophylactic antibiotic duration also differed across diagnostic categories (*F* = 7.58, *P* < .001). In the mixed‐effects infection model, clustering corrections remained significant, and diagnostic category effects continued to lose significance when patient‐level factors were considered. Effect sizes were smaller in the sensitivity analysis, but the direction and statistical significance of all key findings remained unchanged.

## Discussion

Analysis of 429 pediatric tracheostomy patients revealed substantial variation in antibiotic utilization by diagnostic category. Variation that reflects institutional prescribing patterns rather than underlying infection risk. This process‐level assessment identifies high‐variability diagnostic groups as targets for stewardship intervention, with potential downstream effects on length of stay and antibiotic‐associated complications.

Cardiac patients received 10.6 additional days of antibiotics despite having one of the lowest bacterial infection rates (17.8%), indicating that diagnostic categories serve as implicit risk modifiers in clinical decision‐making. This inverse relationship between antibiotic utilization and infectious complications suggests that prescribing patterns are influenced more by institutional practice than by actual clinical risk. When individual patient factors are included in mixed‐effects models, diagnostic differences are no longer significant. Patient‐specific characteristics are therefore more predictive of infection risk than broad diagnostic categories.

The increased antibiotic exposure among young adults likely reflects differing pathology with older patients being afflicted with sepsis, severe respiratory infections, and global developmental delays. In contrast, infants predominantly require tracheostomy for prematurity‐related respiratory failure.

Variation in prophylactic antibiotic duration across diagnostic categories likely reflects uncertainty between evidence‐based guidelines and perceived patient vulnerability. Neuromuscular patients received the longest prophylactic courses, despite limited supporting evidence. The underlying pathophysiology may be associated with increased infectious risk, which can lead to antibiotic utilization that inadvertently undermines stewardship efforts.

Our culture analysis revealed interesting insights about infection surveillance in this population. The all‐cause infection rate of 82.7% (any positive culture) was misleading, as it included viral detections (44.8% of cultures) and predetermined positive bacterial respiratory panels (16.0%). True bacterial infections occurred in only 19.6% of cultures. Additionally, the median time to bacterial infection (~14 days posttracheostomy) suggests that most infections requiring antibiotics are secondary, rather than surgical site, complications, which challenges traditional perioperative antimicrobial paradigms.

Viral detection cascade analysis showed that 11.4% of bacterial cultures were obtained within 48 hours of positive viral results, indicating that viral infections frequently prompt bacterial diagnostic workups. This cascade effect varied by diagnostic category, with congenital condition patients exhibiting the highest rates (17.9%). This ordering pattern may contribute to unnecessary antibiotic initiation when cultures return positive for colonizing organisms rather than true pathogens. Standardized protocols addressing when bacterial cultures are warranted following viral detection represent a discrete stewardship target.

The mixed‐effects model demonstrated good clinical utility with strong discrimination (AUC 0.755) and excellent calibration across risk levels. The patient‐level heterogeneity showed that individual patient factors were more predictive of bacterial infection risk than diagnostic categories or patient age. These findings suggest that prescribing decisions are primarily tailored to individual patients, and the prolonged length of stay increases the risk of infections. From a quality improvement perspective, the observed variability in antibiotic utilization represents a measurable process indicator. Cardiac patients’ high antibiotic exposure despite low infection rates suggests opportunity for standardization without compromising outcomes. Pareto analysis (Figure 1) further supports this prioritization: cardiac and respiratory categories together account for approximately 70% of total antibiotic exposure, suggesting that stewardship process improvement efforts focused on the cardiac diagnostic category may yield the greatest impact. We acknowledge that the lower observed infection rate among cardiac patients may partly reflect a protective effect of their higher antibiotic exposure, a confounding‐by‐indication relationship that cannot be resolved in retrospective analysis. However, the mixed‐effects model's attenuation of diagnostic category effects when accounting for patient‐level factors suggests that prescribing patterns are driven more by institutional practice norms than by a demonstrable treatment effect.

These findings highlight opportunities for stewardship approaches that target both diagnostic‐specific patients and viral‐bacterial culture cascades. The antibiotic utilization differences we observed indicate that clinical complexity amplifies treatment biases. Recent evidence also shows that despite these proclivities, targeted stewardship interventions can be effective in complex pediatric populations.^27^, ^28^, ^29^ Educational interventions targeting specific misconceptions about infection risk across diagnostic categories, coupled with decision support tools that incorporate individual patient risk factors rather than diagnostic categories, may help align prescribing with actual clinical outcomes. Furthermore, protocols to reduce unnecessary bacterial culture ordering following viral detections could decrease healthcare costs and inappropriate antibiotic initiation.

Our findings align with recent work by Steuart et al, who reported high antibiotic utilization rates in pediatric tracheostomy patients.^24^ However, their study focused on longitudinal outpatient care, while our analysis examined perioperative prescribing during index hospitalization, revealing diagnostic‐specific variations not previously described. Additionally, our mixed‐effect model, addresses patient‐level grouping while examining true infection risk from provider prescribing bias.

Lastly, determining which patients warrant posttracheostomy antibiotics requires prospective studies correlating prescribing decisions with clinical outcomes; work this baseline assessment enables.

### Limitations

As a retrospective, descriptive baseline study, our intent was not definitive epidemiology but rather to provide the groundwork for iterative quality improvement. The hierarchical nature of our data, with multiple cultures per patient over prolonged length of stays, required complex statistical modeling that may not capture all relevant patient‐level confounders. Selection bias likely influences findings since tracheostomy placement decisions vary by institutional protocols, potentially creating systematic differences our covariates do not capture.

Our culture classification system relies on component type coding that may not perfectly distinguish bacterial infections from colonization in all cases. Antibiotic utilization may be underestimated due to incomplete capture outside our institution during transfers. The ICD‐10 diagnostic categorization potentially misclassifies patients with multiple complex conditions into single categories. Temporal confounding from evolving stewardship practices during our 10‐year study period may systematically differ across diagnostic groups, though our sensitivity analysis suggests findings are robust.

Additional limitations include: the timing of resistant organism detection suggests hospital acquisition rather than baseline colonization, though absence of preoperative cultures precludes definitive distinction. Specifically, pediatric tracheostomy patients are often sedated and paralyzed in the initial postoperative period, which impairs secretion mobilization and may predispose to pneumonia independent of new organism acquisition. Organisms such as MRSA and *P. aeruginosa* detected in the postoperative period could represent unmasking of pre‐existing colonization rather than de novo acquisition, and without preoperative cultures this distinction cannot be made. This ambiguity further supports the need for stewardship approaches that emphasize clinical context over culture‐driven prescribing. Antibiotic indication was not available; courses could not be definitively attributed to respiratory concerns versus concurrent procedural prophylaxis. Given our aim to describe overall antibiotic utilization, we did not assess antibiotic–culture concordance (eg, organism‐ or susceptibility‐directed therapy). Concurrent surgical procedures that may have prompted antibiotic administration were not captured; and nonrespiratory culture sites (blood, urine) were not examined.

### Generalizability

These findings may not be generalizable to community hospitals or institutions with different patient populations, stewardship infrastructures, or tracheostomy placement criteria. The observed patterns likely reflect tertiary care practices and may differ in settings with less complex populations or different institutional cultures. Healthcare system factors significantly influence prescribing patterns and may limit applicability to different practice environments. However, the mixed‐effects modeling approach demonstrated here could be applied in other institutions to identify local prescribing biases and patient‐specific risk factors.

### Future Directions

Effective antibiotic stewardship must address both clinical protocols and decision frameworks clinicians use to assess risk in pediatric tracheostomy patients. Future research should evaluate diagnostic‐specific stewardship interventions targeting prescribing variation while maintaining clinical effectiveness. Prospective studies examining the impact of individual patient risk scoring, rather than diagnostic category‐based approaches, on antibiotic utilization and clinical outcomes are needed.

The viral detection cascade phenomenon also warrants further investigation. Decision support tools that incorporate mixed‐effects model predictions could help clinicians make more individualized prescribing decisions. Prospective multi‐center studies would enhance generalizability and assess how institutional factors modify the relationship between diagnostic category and antibiotic utilization, while validating the mixed‐effects modeling approach across different healthcare systems.

## Conclusions

Antibiotic utilization in pediatric tracheostomy patients varies substantially by diagnostic category independent of infection risk. This variability represents a process measure amenable to stewardship intervention, with potential to reduce unnecessary exposure, length of stay, and antibiotic‐associated complications.

## Author Contributions

**Romaine F. Johnson,** study concept and design, data interpretation, manuscript drafting, critical revision, final approval; **Saudamini Lele,** study concept and design, data acquisition, statistical analysis, data interpretation, manuscript drafting, critical revision, final approval; **Alyssa C. Chapel,** study concept and design, data interpretation, manuscript drafting, critical revision, final approval; **Cynthia S. Wang,** study concept and design, data interpretation, manuscript drafting, critical revision, final approval; **Yann‐Fuu Kou,** study concept and design, data interpretation, manuscript drafting, critical revision, final approval; **Stephen R. Chorney,** study concept and design, data interpretation, manuscript drafting, critical revision, final approval.

## Disclosures

### Competing interests

Stephen R. Chorney reports educational consulting for Smith and Nephew. All other authors declare no conflicts of interest.

### Funding source

None.

## Supporting information

Abx_Stewardship_Statistical_Code.
