# Supplementary material for: Antibiotic Use and Infection Patterns in Pediatric Tracheostomy Patients During Index Hospitalization
Source: OTO Open. 2026 Aug 17;10(3):e70290. doi: 10.1002/oto2.70290 (PMC13479472; doi:10.1002/oto2.70290)
Supplement: Supplementary file 1 [file OTO2-10-e70290-s001.docx]

/*==============================================================================

PROJECT: Antibiotic Use and Infection Patterns in Pediatric Tracheostomy Patients

TITLE: Complete Statistical Analysis - Updated with Bacterial Infection Modeling

AUTHORS: Romaine F. Johnson, MD MPH

DATE CREATED: August 10, 2025

LAST MODIFIED: August 23, 2025

DESCRIPTION:

This do-file contains all statistical analyses for the pediatric tracheostomy

antibiotic stewardship study. Updated to include bacterial infection analysis,

patient clustering corrections, and viral detection cascades.

DATASETS REQUIRED:

- Abx Stewardship.dta (main patient data, n=429)

- abx stewardship duration.dta (antibiotic courses, n=3,303)

- abx stewardship culture.dta (culture data, n=38,030)

OUTPUTS:

- Table 1: Demographics and Clinical Characteristics

- Table 2: Antibiotic Utilization by Diagnostic Category

- Table 3: Multivariable Regression Results

- Table 4: Bacterial Infection Outcomes by Diagnostic Category (Updated)

- Table 5: Mixed-Effects Logistic Regression - Bacterial Infection Predictors

- Sensitivity Analysis Results

- Model Validation Plots

==============================================================================*/

// Set up environment

clear all

set more off

version 17

capture log close

// Set working directory (modify as needed)

cd "/path/to/your/data"

// Start log

log using "tracheostomy_analysis_`c(current_date)'.log", replace

/*==============================================================================

SECTION 1: DATA PREPARATION AND CLEANING

==============================================================================*/

// Load main patient dataset

use "Abx Stewardship.dta", clear

describe

// Basic data cleaning steps as per Methods

// 1. ICD-10 diagnostic categorization already completed

// 2. Verify diagnostic categories

tab dx_cat, missing

label list dx_cats

// 3. Check for missing data

misstable summarize

count if missing(pat_age, length_of_stay, dx_cat)

// 4. Create analysis variables

gen antibiotic_utilization_rate = (tot_abx_days / length_of_stay) * 100 if !missing(tot_abx_days, length_of_stay)

label var antibiotic_utilization_rate "Antibiotic days as % of total LOS"

// 5. Flag outliers for sensitivity analysis

gen los_outlier = (length_of_stay > 365) if !missing(length_of_stay)

tab los_outlier

/*==============================================================================

SECTION 2: TABLE 1 - DEMOGRAPHICS AND CLINICAL CHARACTERISTICS

==============================================================================*/

display "=== TABLE 1: DEMOGRAPHICS AND CLINICAL CHARACTERISTICS ==="

// Sample size

count

display "Total patients: " r(N)

// Age statistics

summarize pat_age, detail

display "Median age: " r(p50) " years (IQR: " r(p25) "-" r(p75) ")"

// Demographics

tab pat_sex

tab dx_cat

tab aap_age

// Clinical characteristics

summarize length_of_stay icu_days, detail

// Race/ethnicity

tab pat_race

tab pat_ethnicity

/*==============================================================================

SECTION 3: TABLE 2 - ANTIBIOTIC UTILIZATION BY DIAGNOSTIC CATEGORY

==============================================================================*/

// Switch to antibiotic duration dataset

frame create duration

frame change duration

use "abx stewardship duration.dta", clear

display "=== TABLE 2: ANTIBIOTIC UTILIZATION BY DIAGNOSTIC CATEGORY ==="

// Descriptive comparisons across diagnostic categories

dtable, by(dx_cat, test) ///

continuous(tot_abx_days patient_days n_classes antibiotic_utilization_rate) ///

factor(abx_encode_new prophylactic post_surg_abx) ///

nformat(%9.1f mean sd)

// Prophylactic antibiotic analysis

bysort dx_cat: summarize duration if prophylactic == 1, detail

// Statistical test for prophylactic duration differences

oneway duration dx_cat if prophylactic == 1, bonferroni

// Visualization of prophylactic duration

graph bar (mean) duration if prophylactic == 1, over(dx_cat) ///

title("Mean Prophylactic Antibiotic Duration by Diagnosis") ///

ytitle("Days") ///

bar(1, color(navy)) ///

blabel(bar, format(%9.2f))

graph export "prophylactic_duration_by_diagnosis.png", replace

/*==============================================================================

SECTION 4: TABLE 3 - MULTIVARIABLE REGRESSION ANALYSIS

==============================================================================*/

display "=== TABLE 3: MULTIVARIABLE REGRESSION ANALYSIS ==="

// Primary multivariable model

regress tot_abx_days i.aap_age i.dx_cat length_of_stay, robust

// Store results for comparison

estimates store main_model

// Display R-squared and model fit

display "R-squared: " e(r2)

display "Number of observations: " e(N)

/*==============================================================================

SECTION 5: CULTURE DATA PREPARATION AND BACTERIAL INFECTION ANALYSIS

==============================================================================*/

// Switch to culture dataset

frame create culture

frame change culture

use "abx stewardship culture.dta", clear

display "=== CULTURE DATA PREPARATION ==="

// Create comprehensive infection classification

gen infection_type_detailed = .

replace infection_type_detailed = 1 if component_type==1 // Viral detection

replace infection_type_detailed = 2 if component_type==2 // Bacterial respiratory panel

replace infection_type_detailed = 3 if component_type==3 & infection_status==1 // True bacterial infection

replace infection_type_detailed = 4 if component_type==3 & infection_status==0 // Negative bacterial culture

label define inf_types 1 "Viral detection" 2 "Bacterial panel positive" 3 "Bacterial culture positive" 4 "Bacterial culture negative"

label values infection_type_detailed inf_types

label variable infection_type_detailed "Detailed infection classification"

// Create bacterial-only infection variable for main analysis

gen bacterial_infection_only = (infection_type_detailed==3)

label variable bacterial_infection_only "True bacterial infection (excludes viral)"

label define yesno 0 "No" 1 "Yes"

label values bacterial_infection_only yesno

// Identify viral detection cascades

sort mrn result_time

by mrn: gen viral_detection_prior = (infection_type_detailed[_n-1]==1 | infection_type_detailed[_n-2]==1) if infection_type_detailed==3

label variable viral_detection_prior "Bacterial culture within 48h of viral detection"

label values viral_detection_prior yesno

// Display infection type distribution

tab infection_type_detailed

tab component_type infection_status, row

/*==============================================================================

SECTION 6: TABLE 4 - BACTERIAL INFECTION OUTCOMES BY DIAGNOSTIC CATEGORY

==============================================================================*/

display "=== TABLE 4: BACTERIAL INFECTION OUTCOMES (UPDATED) ==="

// Bacterial infection rates by diagnostic category (corrected analysis)

tab dx_cat bacterial_infection_only, row chi2

// Time to culture analysis

summarize time_to_culture if bacterial_infection_only == 1, detail

// Viral cascade analysis

tab viral_detection_prior dx_cat if bacterial_infection_only == 1, col chi2

// Resistant organism analysis (bacterial cultures only)

tab dx_cat mrsa_post if bacterial_infection_only == 1, row chi2

tab dx_cat pseudomonas_post if bacterial_infection_only == 1, row chi2

// Culture timing analysis

gen time_cat = 1 if time_to_culture < 0

replace time_cat = 2 if time_to_culture >= 0 & time_to_culture <= 7

replace time_cat = 3 if time_to_culture > 7 & time_to_culture <= 30

replace time_cat = 4 if time_to_culture > 30 & !missing(time_to_culture)

label define time_cats 1 "Pre-operative" 2 "≤7 days" 3 "8-30 days" 4 ">30 days"

label values time_cat time_cats

tab time_cat bacterial_infection_only, row chi2

/*==============================================================================

SECTION 7: TABLE 5 - MIXED-EFFECTS LOGISTIC REGRESSION

==============================================================================*/

display "=== TABLE 5: MIXED-EFFECTS LOGISTIC REGRESSION - BACTERIAL INFECTION ==="

// Check clustering extent

bysort mrn: gen culture_count = _N

summarize culture_count, detail

display "Average cultures per patient: " r(mean)

display "Max cultures per patient: " r(max)

// Mixed-effects logistic regression (accounts for patient clustering)

melogit bacterial_infection_only i.dx_cat i.aap_age time_to_culture || mrn:, or

// Store results

estimates store mixed_effects_model

// Compare to standard logistic (for sensitivity)

logit bacterial_infection_only i.dx_cat i.aap_age time_to_culture, vce(cluster mrn) or

estimates store clustered_logit

// Model comparison

lrtest mixed_effects_model clustered_logit

/*==============================================================================

SECTION 8: MODEL VALIDATION AND DIAGNOSTICS

==============================================================================*/

display "=== MODEL VALIDATION ==="

// Use mixed-effects model for validation

estimates restore mixed_effects_model

// Generate predicted probabilities

predict prob_me, mu

// Calibration analysis

egen prob_decile = xtile(prob_me), nq(10)

preserve

collapse (mean) obs_rate=bacterial_infection_only pred_rate=prob_me, by(prob_decile)

// Calibration plot

twoway (scatter obs_rate pred_rate) (line pred_rate pred_rate), ///

title("Model Calibration Plot") ///

subtitle("Mixed-Effects Logistic Regression") ///

xlabel(0(0.1)1) ylabel(0(0.1)1) ///

xtitle("Predicted Probability") ytitle("Observed Rate") ///

legend(order(1 "Observed" 2 "Perfect Calibration") pos(11))

graph export "calibration_plot.png", replace

restore

// Discrimination analysis (AUC)

roctab bacterial_infection_only prob_me

local auc = r(area)

display "Area under ROC curve: " %5.3f `auc'

// Generate ROC curve

roctab bacterial_infection_only prob_me, graph ///

title("ROC Curve - Bacterial Infection Prediction") ///

subtitle("AUC = " %5.3f `auc')

graph export "roc_curve.png", replace

/*==============================================================================

SECTION 9: SENSITIVITY ANALYSIS

==============================================================================*/

frame change duration

display "=== SENSITIVITY ANALYSIS: EXCLUDE LOS >365 DAYS ==="

// Create sensitivity analysis sample

local los_cutoff = 365

gen sensitivity_sample = (length_of_stay <= `los_cutoff') if !missing(length_of_stay)

tab sensitivity_sample

// Display sample sizes

count

display "Original sample size: " r(N)

count if sensitivity_sample == 1

display "Sensitivity analysis sample size (LOS ≤365 days): " r(N)

count if sensitivity_sample == 0

display "Excluded courses: " r(N)

// Repeat main regression analysis in sensitivity sample

regress tot_abx_days i.aap_age i.dx_cat length_of_stay if sensitivity_sample == 1, robust

estimates store sensitivity_model

// Compare models

estimates table main_model sensitivity_model, b(%9.3f) se(%9.3f) stats(N r2)

// Sensitivity analysis for bacterial infection model

frame change culture

gen sensitivity_sample_culture = (length_of_stay <= 365) if !missing(length_of_stay)

melogit bacterial_infection_only i.dx_cat i.aap_age time_to_culture if sensitivity_sample_culture == 1 || mrn:, or

estimates store mixed_effects_sensitivity

// Compare infection models

estimates table mixed_effects_model mixed_effects_sensitivity, eform b(%9.3f) se(%9.3f)

/*==============================================================================

SECTION 10: ADDITIONAL ANALYSES AND FIGURES

==============================================================================*/

frame change duration

// Create additional visualizations

// Box plot of antibiotic utilization by diagnosis

graph box antibiotic_utilization_rate, over(dx_cat) ///

title("Antibiotic Utilization Rate by Diagnostic Category") ///

ytitle("Percentage of LOS on Antibiotics") ///

note("Boxes show median and IQR; whiskers show range")

graph export "utilization_rate_boxplot.png", replace

// Scatter plot: LOS vs total antibiotic days

scatter tot_abx_days length_of_stay, by(dx_cat) ///

title("Total Antibiotic Days vs Length of Stay") ///

xtitle("Length of Stay (days)") ///

ytitle("Total Antibiotic Days")

graph export "antibiotics_vs_los_scatter.png", replace

// Culture timing visualization

frame change culture

hist time_to_culture if bacterial_infection_only == 1 & time_to_culture >= 0, ///

title("Distribution of Time to Bacterial Infection") ///

subtitle("Days from Tracheostomy to Positive Culture") ///

xtitle("Days") ytitle("Frequency") ///

bin(20)

graph export "time_to_infection_hist.png", replace

/*==============================================================================

SECTION 11: SUMMARY STATISTICS FOR MANUSCRIPT

==============================================================================*/

display "=== SUMMARY STATISTICS FOR MANUSCRIPT ==="

frame change default

// Key numbers for abstract and results

count

display "Total patients: " r(N)

summarize pat_age, detail

display "Median age: " r(p50) " years (IQR: " r(p25) "-" r(p75) ")"

tab dx_cat

tab pat_sex

frame change duration

count

display "Total antibiotic courses: " r(N)

frame change culture

count

display "Total cultures: " r(N)

// Original infection rate (including viral)

count if infection_status == 1

local infections_all = r(N)

count

local total_cultures = r(N)

display "Overall positive culture rate: " %4.1f (`infections_all'/`total_cultures'*100) "%"

// Corrected bacterial infection rate

count if bacterial_infection_only == 1

local bacterial_infections = r(N)

display "True bacterial infection rate: " %4.1f (`bacterial_infections'/`total_cultures'*100) "%"

// Viral detection rate

count if infection_type_detailed == 1

local viral_detections = r(N)

display "Viral detection rate: " %4.1f (`viral_detections'/`total_cultures'*100) "%"

// Cascade analysis summary

count if viral_detection_prior == 1 & bacterial_infection_only == 1

local cascades = r(N)

count if bacterial_infection_only == 1

local total_bacterial = r(N)

display "Bacterial cultures following viral detection: " %4.1f (`cascades'/`total_bacterial'*100) "%"

/*==============================================================================

SECTION 12: DATA EXPORT FOR TABLES

==============================================================================*/

// Export key results for table creation

frame change duration

// Export summary statistics by diagnostic category

preserve

collapse (mean) mean_abx=tot_abx_days mean_los=length_of_stay mean_util=antibiotic_utilization_rate ///

(sd) sd_abx=tot_abx_days sd_los=length_of_stay sd_util=antibiotic_utilization_rate ///

(count) n=tot_abx_days, by(dx_cat)

export excel using "table2_summary.xlsx", firstrow(variables) replace

restore

// Export regression results

estimates restore main_model

esttab using "table3_regression.csv", b(3) se(3) star(* 0.05 ** 0.01 *** 0.001) ///

stats(N r2, fmt(0 3)) replace

// Export bacterial infection results

frame change culture

preserve

collapse (mean) bacterial_rate=bacterial_infection_only viral_cascade=viral_detection_prior ///

(count) total_cultures=bacterial_infection_only, by(dx_cat)

replace bacterial_rate = bacterial_rate * 100

replace viral_cascade = viral_cascade * 100 if !missing(viral_cascade)

export excel using "table4_bacterial_infections.xlsx", firstrow(variables) replace

restore

// Export mixed-effects model results

estimates restore mixed_effects_model

esttab using "table5_mixed_effects.csv", eform b(3) se(3) star(* 0.05 ** 0.01 *** 0.001) ///

stats(N ll, fmt(0 2)) replace

/*==============================================================================

END OF ANALYSIS

==============================================================================*/

// Close log

log close

display "Analysis complete. Check log file for detailed results."

display "Generated files:"

display "- tracheostomy_analysis_`c(current_date)'.log"

display "- prophylactic_duration_by_diagnosis.png"

display "- utilization_rate_boxplot.png"

display "- antibiotics_vs_los_scatter.png"

display "- calibration_plot.png"

display "- roc_curve.png"

display "- time_to_infection_hist.png"

display "- table2_summary.xlsx"

display "- table3_regression.csv"

display "- table4_bacterial_infections.xlsx"

display "- table5_mixed_effects.csv"

display "Key findings:"

display "- Mixed-effects modeling accounts for patient clustering (38,030 cultures from 429 patients)"

display "- True bacterial infection rate: ~20% (vs 83% when including viral detections)"

display "- Model shows good calibration and discrimination (AUC ~0.755)"

display "- Diagnostic category effects largely disappear when accounting for clustering"

display "- Individual patient factors more predictive than diagnostic categories"
